# Harnessing 3D Printing of Plastics in Construction—Opportunities and Limitations

**DOI:** 10.3390/ma14164547

**Published:** 2021-08-13

**Authors:** Aneta Skoratko, Jacek Katzer

**Affiliations:** Faculty of Geoengineering, University of Warmia and Mazury in Olsztyn, 10-720 Olsztyn, Poland; jacek.katzer@uwm.edu.pl

**Keywords:** 3D printing, plastics, reinforcement, additive manufacturing technology

## Abstract

Additive manufacturing has been of increasing interest to the construction industry for the last ten years. The subject of the research is the printing of concrete, metals, and plastics. In their analysis and research, authors have focused on printing plastics. 3D printing of reinforcement of concrete elements made of plastics can significantly improve the efficiency of their erection, reduce the amount of waste, and optimize their shape. In this paper, recent developments in the 3D printing of plastics for construction are reviewed. Various applications were discussed, including unconventional spatial reinforcement (impossible to achieve in a traditional way), printed permanent formwork, etc. The challenges for further research and practical applications of such solutions were also discussed.

## 1. Introduction

Over the last decades, 3D printing has become one of the fastest-growing and promising branches of technology associated with robotics and IT. 3D printers have found widespread use in numerous industries: automotive, medical, construction, and mechanical engineering [1,2,3,4]. Improving printers, printing technology, and materials used for 3D printing is the goal of many research programs around the world. The most current subject of 3D printing research is the printing of concrete, metals, and plastics. The progress of civilization and the need to reduce CO_2_ emissions compels all parties to look for new construction technologies and materials. These new solutions should respond to the growing quantitative and qualitative needs of the construction industry while adhering to the principles of sustainable development [5,6,7,8]. The construction sector needs to reduce its negative impact on the environment and tackle climate change [9]. The need to print materials that perform structural, thermal insulating, or lighting functions in a building lead to the concept of new 3D printing technologies and solutions. All these activities create a new chapter in construction technology [10,11]. 3D printing has the potential to become a highly sustainable technology. Concrete mixes with aggregate derived from recovered construction waste are used for printing concrete, while both natural and artificial filaments are used for printing plastics. Currently, polymers in the form of powder, polymers made from renewable raw materials, resin, monomers, and thermoplastic fiber are used for additive manufacturing [12]. Plastic waste generated during 3D printing (e.g., in the form of generated temporary supports, etc.) can be melted, processed, and also recycled into new filaments. 3D printing technology emerges as a key opportunity to preserve our natural environment [13,14]. The advantage of using 3D printing in construction is also the minimization of “wet” construction processes. As a result, less material waste and dust are generated during the construction process of a building. The achievements of 3D printing technology presented below are associated with the 3D printing of concrete, metals, and plastics [15]. Spatial plastic elements created in FDM technology (fused deposition modeling) were of special interest. This technology is based on building a spatial element with the help of a molten thermoplastic material forced through a nozzle. The nozzle is located under the thermal head with the material (filament) extruder. The arrangement of the filament layers according to the geometry of the designed model enables the realization of the 3D print through the horizontal movement of a thermal head and the vertical movement of a work platform. The layers are placed one after another [16,17]. The applied fresh layer of material solidifies and merges with the previous one. A digital model of an object to be printed is created directly from a digital design (e.g., created using CAD software) [18]. The most popular format for digital models for printing is STL. Charles Hull, an American scientist, is considered to be the founder of this 3D printing technology. He patented the stereolithography technique by introducing the STL format [19]. The additive way of arranging the material allows for complete architectural freedom by obtaining very complex forms and bold spatial designs [20,21]. Several types of materials are used by the FDM method(Fused Deposition Modeling): ABS(akrylonitrylo-butadieno-styren), polycarbonate, PLA(polylactide), nylon, etc. However, the most common are ABS and PLA.

**Figure 1:**
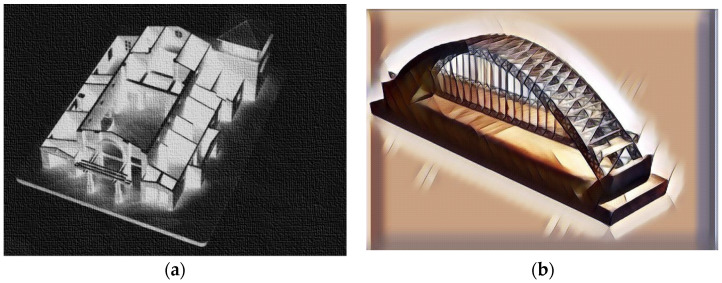
Mock-ups of (**a**) a public building; (**b**) a bridge created by 3D printing. Images created by A. Skoratko (inspired by [23]).

Currently, 3D printing is harnessed in all stages of the construction process. During the design stage, mock-ups are made that show the object in accordance with the architectural design. Visualization helps to see details, the arrangement of windows and doors, stairs, or the arrangement of load-bearing and partition walls. Thanks to this, the investor can change the concept before the start of the construction phase. Mock-ups are also used to design engineering structures such as footbridges, bridges, and viaducts. It is also a way of verifying the correctness of the project [22]. Examples of mock-ups are shown in Figure 1.

## 2. 3D Printing of Concrete and Metals

**Figure 2:**
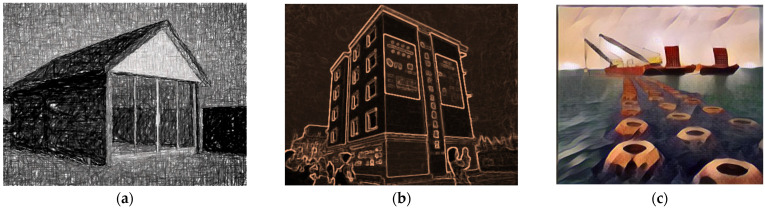

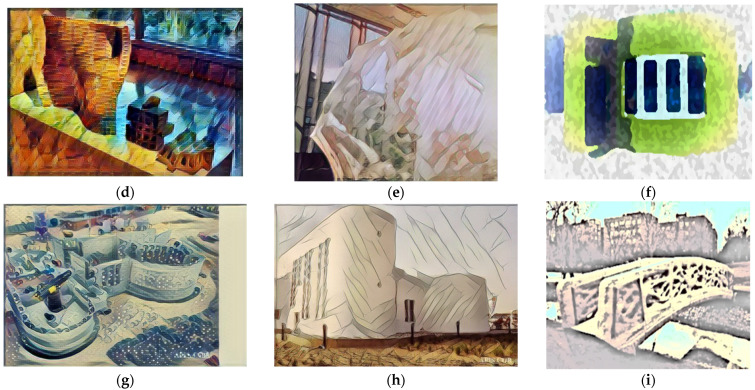
Examples of 3D printed concrete buildings and structures: (**a**) one of the first houses; (**b**) multi-story house; (**c**) concrete artificial reefs; (**d**) PolyBricks blocks; (**e**) construction modeled on the work of silkworms; (**f**) a house made according to the design of Apis Cor; (**g**) the construction phase of the largest building; (**h**) the largest 3D-printed building; (**i**) Castilla-La Mancha pedestrian bridge. Images created by A. Skoratko (inspired by [34,36,41,42]).

The technology of 3D printing concrete buildings and structures is developing the fastest and gives almost unlimited possibilities. Contour crafting and concrete printing technology are most often used. Manufacturers strive to increase the environmental performance of the construction process. Thus, the material used for printing is often a concrete mix consisting of cement reinforced by glass fiber and aggregate that comes from recovered construction waste (debris from demolished buildings). The printed concrete layers are clearly separated from each other, which adversely affects the aesthetics of a wall, but only at the shell stage. On the construction site, rails adapted to the movement of the printer are placed, then, based on a computer project, the object is made in its shell condition. The nozzles pump the concrete onto the construction elements. It is also possible to produce prefabricated elements in the factory. To create a building in printing technology, one needs 50–70% less time than in the case of traditional construction [24]. The country that develops the most dynamically in this direction is China [25]. The leading company is WinSun. In 2014, 10 houses with an area of 200 m^2^ were printed by WinSun in one day (Figure 2a). In 2015, a four-story residential building (Figure 2b) and an exclusive villa with an area of 1100 m^2^ were built [26,27]. Another achievement of the Chinese is the erection of a house from 3D printed elements within 3 h. The house was to be fireproof and resistant to shocks with a strength of 9° on the Richter scale [28]. The first 3D-printed office building is located in Dubai, and the construction of the first high-rise building was planned there in 2017 [29]. In Zurich, sand 3D printing was used to make a slab ceiling, that weighed 15 tons and measured 80 m^2^ [30]. In 2017, the American company Apis Cor presented the first residential house with an area of 38 m^2^, which was built in just 24 h in Russia (Figure 2f) [31]. It was created directly on the construction site and is characterized by an unusual shape. It proves the possibilities of designing and erection of unique geometries thanks to 3D printing. In 2018, the first French family moved into a 3D printed, five-room house, with an area of 95 m^2^. The skeleton of the walls is made of polyurethane foam and the voids are filled with concrete. The projects related to 3D printing residential houses are also carried out in Dubai and the Netherlands [32,33]. Also in Poland, the first building made of concrete mix using a printer was built in 2019. The printing process lasted 13 h and the house had an area of 7 m^2^. At the end of 2019, in Dubai, the largest 3D printer building in the world was completed (Figure 2g,h). The building is 9.5 m high, has an area of 640 m^2^, and was made of a mixture that came from the recycling of construction waste. It took 3 weeks to complete the structure. The process of 3D printing required only three employees to be involved. This proves that 3D printers are the future of construction, but still require human presence [34,35]. 3D printing is also used to produce artificial reefs. A company called Reef Arabia has developed a plan to restore offshore rock with concrete layers. Artificial reefs (Figure 2c) have already been delivered to Australia [36]. An interesting project is the 3D printing of PolyBricks ceramic blocks (Figure 2d), which would contribute to masonry without mortar. The elements are designed to be connected with each other thanks to special tabs. They were made of a raw material similar to powdered clay. A very interesting building was erected at the Massachusetts Institute of Technology on the basis of a design by Neri Oxman; a pavilion in which the 3D-printed skeleton is similar to the production of silk by silkworms (Figure 2e). Their movement was reproduced by the printing device. This was to create the possibility of creating prototype objects using a large number of small robots to avoid large-size installations and overhead traveling cranes [36,37].

Apart from 3D printing buildings, it is possible to create pedestrian bridges, emergency shelters, military bunkers, molds, and formworks for load-bearing elements and the reproduction of historical building elements, etc. [38,39]. The first 3D printed bridge is the Castilla-La Mancha pedestrian bridge in Madrid (Figure 2i). It is 1.75 m wide with a span of 12 m. It was erected using melted concrete powder and polypropylene reinforcement [40].

**Figure 3:**
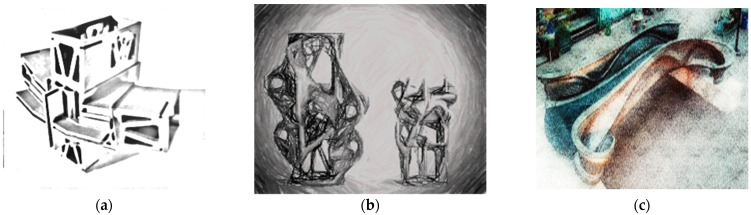
Examples of 3D printed metal elements: (**a**) aluminum node; (**b**) optimized nodes; (**c**) bridge MX3D. Images created by A. Skoratko (inspired by [43,45,46]).

The 3D printing of metals can also be used in construction by combining printed molds with traditional structural shapes that create hybrid structures. It is also used to create internal stiffeners, openings, non-prismatic sections or connection nodes, and metal structural sections [43]. The load-bearing capacity of beam truss structures was thoroughly tested [44] and the achieved results are very promising. Early attempts of harnessing 3D printing of metals were focused only on small-scale components, while work is currently underway to build a full-size pedestrian bridge. Metal additive manufacturing techniques include powder bed fusion (PBF), targeted energy deposition (DED), sheet and mold lamination, and electrochemical additive manufacturing. Examples of printed metal elements are shown in Figure 3.

## 3. 3D Printing of Plastics for Construction

When manufacturing structurally optimized (with non-standard shapes) concrete elements using the traditional casting methods, the prepared formwork is destroyed during dismounting [47,48]. As the 3D printing method is not limited by any kind of formwork, it provides designers with greater design flexibility in this respect [20]. Formworks for structural concrete elements, which account for 40% of the total budget for concrete works, can be avoided during the construction process, ultimately shortening the project schedule without incurring additional costs [3]. Thanks to the 3D printing technology, the designing of structures will not be limited to simple formwork and a collection of monotonous prefabricates [5,49]. The construction industry is able to use large amounts of recycled plastic in the form of printed formwork. Currently, the possibility of using waste plastic for the production of filament is in the scope of the research team of Prof. M. Hinge at Aarhus University in Denmark [50]. The use of plastic reinforcements instead of steel or plywood ones is a very tempting vision from the ecological point of view. The industry has made great efforts to completely eliminate the need for traditional formwork. Although these changes give hope for the possibility of building surfaces of any shape completely waste-free, there are some limitations and difficulties associated with such systems.

**Figure 4:**
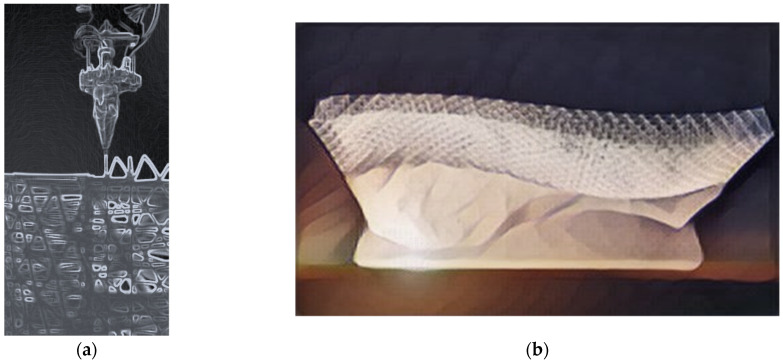

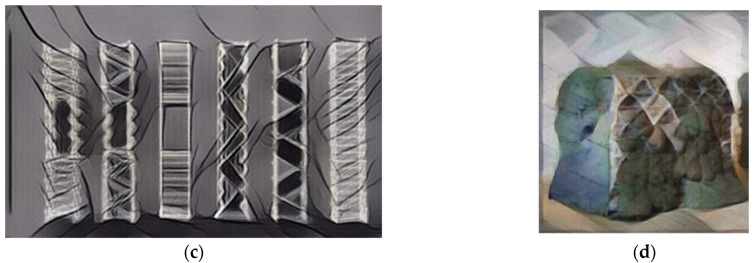
Mesh molds (**a**) polymer extrusion; (**b**) first mesh prototype filled with concrete; (**c**) various mesh models; (**d**) test cubes after casting. Images created by A. Skoratko (inspired by [51,52]).

Hack and Lauer [51] describe the research they undertook to develop a mesh mold as an alternative to conventional formwork based on flexible in situ production. This provides an opportunity to not be limited to geometric simplification, standardization, and repetition. The polymers were freely extruded in 3D space, precisely controlled by a robot to prepare the necessary meshes and free the formwork from geometric constraints. The use of thermoplastic polymers, as in conventional 3D printers, enables control over the hardening of the material. The possibility of cooling during the extrusion process provides the option of 3D extrusion and creating a skeleton structure (Figure 4a). Then, concrete is poured over this formwork, and it is rubbed by hand in order to smooth the surface (Figure 4b). Moreover, the mesh densities are generated depending on the various forces acting on the structure (Figure 4c). More interestingly, the presence of the mesh increases the tensile strength of the concrete, making it eventually a possible replacement for conventional steel reinforcement [52]. Designing meshes of any shape not only allows local adaptation to the stresses or existing curvatures but also allows the integration of cavities for lighter porous structures.

The main goal of the research by Hack et al. [52] was to prove that spatial extrusion can be applied to complex surfaces. A stiff fresh concrete mix was not poured from the top, but pressed from the outside through the mesh, successively compacting the interior. The strategy turned out to be effective but labor-intensive. After applying 35 kg of fresh concrete mix, no deformation of the mesh was found. With this solution, the flow of concrete should be optimized. The meshes should not be too thin for the concrete to leak out, but also not too dense for the concrete to cover the structure evenly without leaving any internal voids. The same type of mesh was also tested, but with concrete top filling. The aim was to obtain the best relationship between the viscosity of the concrete and the mesh size. Cubes with an edge of 15 cm (Figure 4d) and with different dimensions of the holes were examined. Two mixtures were used, one with the addition of fiber and the second without fiber. The fibers caused clogging in the dense internal structure. The fiber-free mixture performed best with a 19 cm descending flow as measured with a Haegermann mini-cone. Overall, the internal structure was found to be too dense. It prevented the concrete from flowing evenly around the plastic spatial mesh. The third series of tests was to check the structural integrity of the meshes during the pouring process. A series of test cubes with different numbers of holes and different hole sizes were placed at the bottom of one 15 × 15 × 15 cm pressure column. The column was filled with concrete from the top. Experiments showed that none of the meshes cracked under the weight of the concrete.

As demonstrated, it is possible to use 3D-printed plastic elements as an unconventional reinforcement [53,54,55]. The formwork would be used for the traditional casting of concrete structural elements. The printed form with concrete would form a composite with high flexural strength.

**Figure 5:**
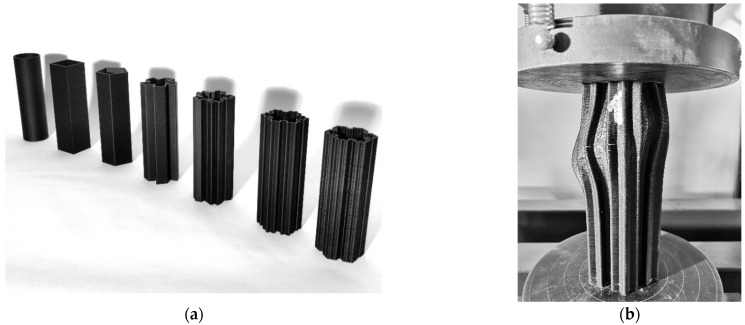
3D printed column formworks (**a**); deformation of a concrete-plastic column during compression (**b**) Images created by A. Skoratko.

Katzer and Skoratko [53] presented the concept of plastic-concrete columns. Seven different sections of 3D-printed formwork made of plastics were prepared (Figure 5a). The selected shapes corresponded to the three groups of most frequently encountered, rarely encountered, and impossible to realize (based on fractals) cross-sections. All columns were characterized by a constant height of 160 mm and a constant cross-sectional area of 1600 mm^2^. They were filled with cement mortar and tested after 28 days. The only variable parameters were the shape of the cross-section and the amount of material used to create a given shape. The relationship between the load and deformation of columns was investigated. Concrete-plastic columns were characterized by a quasi-plastic behavior and were eventually destroyed. The more complicated cross-section, the more quasi-plastic characteristics were achieved. For the group of formwork based on fractals, the formwork did not crack during the loading process but was deformed (Figure 5b). Such behavior would contribute to the detection of the failure of the structure—the signaled failure. In addition, such properties may be of key importance in the event of an earthquake or explosion, allowing the evacuation of people. The columns with cross-sections impossible to be made with the use of traditional formwork withstood the greatest deformations while maintaining a significant part of the maximum load. The authors noticed that the size of the plastic surface, taking it into account to determine the compressive strength, may have a significant influence on the result, especially in the case of fractal-based cross-sections. Compressive strength is not an ideal parameter for determining mechanical properties due to the unconventional behavior of the tested columns. Therefore, the authors, on the basis of the load-deformation relationship, calculated the required energy that is needed until the element is destroyed.

**Figure 6:**
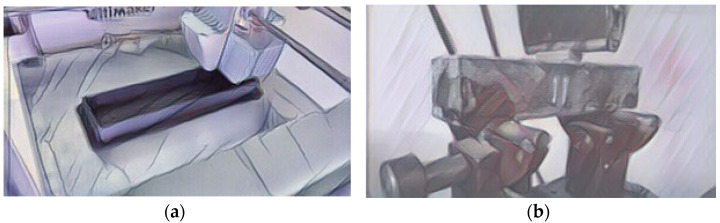
3D printed beam formwork (**a**); flexural strength test of an element with 3D printed formwork (**b**). Images created by A. Skoratko (inspired by [54]).

Katzer and Szatkiewicz [54] proposed a 3D printed plastic formwork for making beams. The samples were made in two phases. Firstly, the formwork was printed (Figure 6a), and then it was filled with cement matrix. Additionally, the formwork had ribs (from 0 to 3), characterized by height from 0 to 15 mm. The geometry of the ribs was very basic. They were to play the role of rebars. A constant dimension was the thickness of the outer walls, ribs, and outer dimensions. The reference point was samples made from the cement matrix itself. After the concrete was poured, the plastic formwork remained in its place and served as a reinforcement for the concrete element. The specimens were subjected to a flexural strength test with one central point of loading (Figure 6b). The force was only applied to the surface of the cement matrix.

The results obtained by the authors showed that the behavior of the beams with the formwork is quasi-plastic. The mechanical behavior was compared to that of ferro-cements and concretes reinforced with dispersed reinforcement. The presence of additional ribs was found to influence the flexural strength. The highest strength was achieved with three ribs each 15 mm high. Formwork-matrix samples without ribs are able to achieve almost twice the strength value of samples made of the cement matrix itself. On the other hand, samples with formwork and ribs are able to achieve four times the strength of the plain element. 

**Figure 7:**
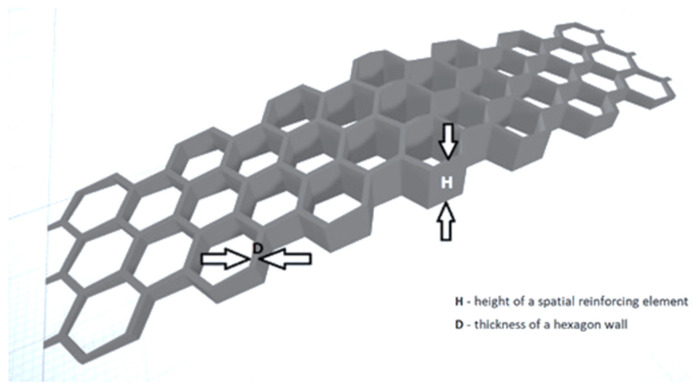
General view of the designed spatial reinforcing element. Image created by A. Skoratko (inspired by [55]).

Other work by Katzer and Szatkiewicz [55] investigated the flexural characteristic of samples reinforced with 3D printed hexagonal elements based on hexagonal geometry. This geometry was selected for the spatial reinforcement based on proven rigid mechanical properties [56]. Hexagonal geometry was also used to create beam lattice structures [44]. The printed elements were placed into steel molds and then filled with cement mortar. The reinforcing element had a triangular cross-section along the length of the beam (Figure 7). The spacing of the hexagons and their sizes were kept constant. The variable parameters were: height in the middle of the span *H* and wall thickness *D*. The authors designed four different heights and thicknesses.

The flexural strength of a beam increased with the increasing wall thickness and height *H* of a reinforcing plastic element. The appropriate selection of these two parameters allowed to achieve the value of the maximum force equal to 184% of the characteristics of pure mortar. It has been proven that it is possible to effectively reinforce concrete elements with the appropriate shape and size of a 3D printed reinforcing element.

**Figure 8:**
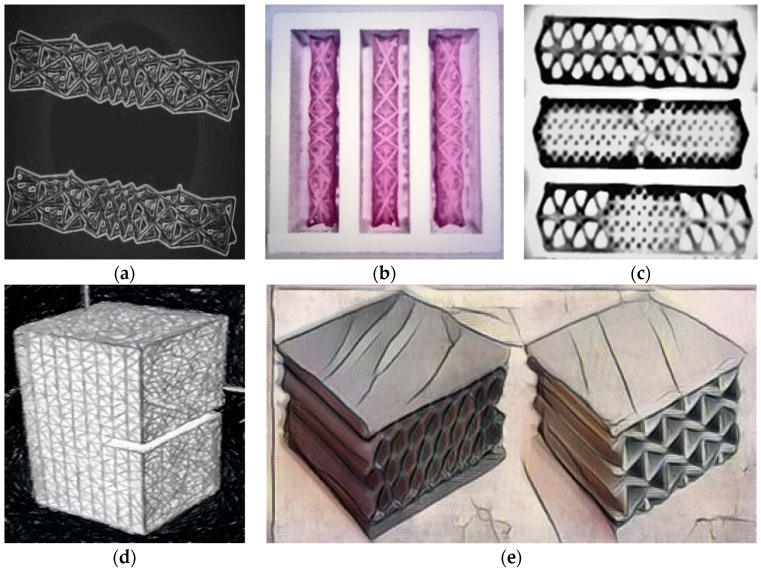
(**a**) functionally graded octet lattice structures; (**b**) printed specimens in polystyrene molds; (**c**) printed reinforcement with different triangle patterns; (**d**) printed specimens with a notch for tensile testing; (**e**) examples of 3D printed PLA sandwich panels. Images created by A. Skoratko (inspired by [57,58,59,60]).

The 3D printing technology was also used to reinforce cement composites with printed polymer network structures [57]. The structure of the designed polymer reinforcement can be adapted to the bending moment and tensile stress distribution. The authors [58] designed and printed four different lattice structures (Figure 8a), used to reinforce the cement mortar. The samples were subjected to four-point bending. The printed elements were placed inside the polystyrene molds (Figure 8b). During the tests, it was shown that the samples reinforced with a 3D-printed mesh made of polymer material showed plastic characteristics. The brittle fracture of the conventional cement mortar was changed to ductile fracture. The toughness of the composites, with respect to conventional cementitious material, increased in terms of ductile failure strength and overall work required to break. It has been found that the appropriate functional adaptation of the reinforcements increases flexural flexibility while using less reinforcement.

The same researchers in another work [60,61,62] 3D-printed a polymer reinforcement. The reinforcing meshes were to be an alternative to creating SHCC(Strain-Hardening Cement-Based Composites). During the tests, it was proved that the appropriate design of the reinforcing mesh makes it possible to obtain cement composites with enhanced deformation and deflection characteristics. The multiple cracking of the traditional SHCC was also observed in the case of elements with 3D printed polymer reinforcement. Three different reinforcements differentiated by geometric triangle patterns were tested. The shape of printed reinforcing elements is presented in Figure 8c. Additionally, different roughness of the printed reinforcements was used. The samples were subjected to four-point bending and uniaxial tensile loadings. Due to the applied reinforcements, the samples could undergo greater deformations and achieve higher tensile strength. Some mesh designs enabled the creation of composites with SHCC properties.

Gödek et al. [63] created honeycomb-shaped grids of two thicknesses, 1 mm and 2 mm, printed from PLA. The reinforcements were flat, and also with protrusions. The samples were subjected to three-point bending. The thicker specimens with protrusions showed hard deformation. The tabs slightly improved the bending strength (more evident in the thicker samples). Both the bulge and the thickening improved the deflection characteristic and the toughness value.

The uniaxial tensile strength of disordered 3D-printed lattice structures was also tested (Figure 8d). A comparison was conducted of the experimental and simulation results, including a discussion on the influence of the printing direction. Properties vary depending on the printing direction. It has been shown that the greater the randomness and disorder of the lattice material, the lower the strength, stiffness, and brittleness of these elements [58].

Studies were also carried out to determine the preferred architectural geometry of 3D printed polymer sandwich panels (Figure 8e) [59]. The printed panels were characterized by different cell topologies. The panels were subjected to impact tests to determine their efficiency and ability to absorb energy.

Farina et al. [64] focused their research on printing discrete rebars using Fullcure 720 photopolymer resin. The prepared bars had both a smooth and a rough surface. They were used for three-point bending studies. Three types of samples were compared: reference (without reinforcement), smooth, and rough reinforcement. Reference samples underwent brittle failure, samples with smooth reinforcement, after reaching the peak load, showed a constant, residual load capacity, while samples with rough reinforcement after peak load lost their bearing capacity, and then hardened until reaching the second peak, after which failure took place. The specimens with smooth reinforcement underwent destruction typical for bending and propagation of the central crack until destruction. Shear failure (numerous diagonal cracks) was observed in samples with rough reinforcement. Samples with reinforcement, compared to the reference samples, showed a significant efficiency of energy absorption.

Rebars were also printed using PLA and PETG (Polyethylene terephthalate with glycol) thermoplastic polymers [65]. They were used to reinforce beams made of masonry mortar with dimensions of 200 × 50 × 50 mm. The bars had a rectangular cross-section of 10 × 5 mm and a length of 190 mm. Four pieces were embedded in the sample and subjected to three-point bending tests. The samples with the reinforcement made of PLA showed significantly worse bending behavior in comparison to the samples where PETG was used, in terms of deformation and limit loads. The average flexural strength of PETG samples was almost twice as high, and the deformation of these samples at maximum load reached three times the deformation of PLA samples. A tensile test was also performed for both polymers in question. Both achieved similar strength, while PETG did not suddenly fail and showed extensive elongation. The energy absorbed by the PETG samples was also higher (5 times, compared to the PLA samples).

Key results of the discussed research programs are summarized in Appendix A of this paper.

## 4. Opportunities and Challenges

One can safely state that printing technology offers great opportunities for the future conquest of space. Thanks to the use of this method, instead of transporting the construction elements necessary for the erection of the base, from Earth. Astronauts would be able to use lunar (or Martian) regolith as raw material for 3D printing. Research on this issue is currently being carried out by scientists from the European Space Agency (ESA) in cooperation with the company of the famous architect Norman Foster—Foster and Partners [39,66,67].

A typical concreting process includes a long and sequential chain of tasks from pre-perforation of formwork, transportation, on-site logistics, bending and laying rebar, assembly of formwork, concreting, dismantling, and cleaning of the formwork, right through to surface finishing. The described research and possibilities aim at simplifying the technological process itself [51,68]. It was also forecast that the complex fractal cross-sections of the columns, apart from their artistic value, may have an impact on noise reduction in public spaces [53].

3D printing would minimize the dependence of the construction industry on the human factor. In addition, it would contribute to increasing productivity and improving the health and safety of construction workers. The riskiest or most dangerous works would be conducted using innovative technologies [69,70].

Formwork printed with the use of plastic with internal elements is an opportunity to partially eliminate traditional steel reinforcement. The mechanical characteristics of 3D printed plastics are inferior in comparison to steel. Nevertheless, the innovative internal shape of the formwork and the importance of the lost formwork itself can significantly substitute the weaker mechanical properties of the plastic [54]. One can adjust the reinforcement shape and size to the spacing of stresses in the element.

Laboratory printers designed for the production of small and precise elements are the limitation of 3D printing of plastics for civil engineering applications. Printing with real dimensions formwork and reinforcement is currently not possible. The modulus of elasticity of polymeric materials used for 3D printing is limited. However, thanks to appropriate processing and the use of appropriate printing parameters, their strength can be increased [57]. Adding fiber to a pure polymer matrix may enable the creation of 3D printed plastic elements with higher structural strength [71]. It would be environmentally friendly to use natural fibers together with thermoplastics. This would reduce the emission of gases produced with synthetic fiber. The appropriate selection of natural fiber added to polymers would contribute to the creation of new filaments with various desirable properties [72]. Commercially available 3D printers were created for prototyping purposes (e.g., complicated parts of engines, etc.). Therefore, they were created with a very high precision of printed elements as a key factor. For civil engineering applications, a much larger scale of printed plastic elements is needed, but their precision can be lower (in comparison to mechanical parts) and surfaces rougher. For such “rough” civil engineering precision, filaments produced from the recycling of waste plastics seem a perfect fit.

## 5. Conclusions

Currently, construction is one of the most wasteful industries. The exploitation of additive manufacturing technology could provide a safer, faster, and more economical construction process. Additionally, it would contribute to greater flexibility of an architect’s work. The printing devices can work without interruptions, which guarantees high efficiency. The costs of printing a residential building are lower than in the case of traditional technologies, which will significantly affect the price of flats [7,22]. However, it is difficult to imagine that 3D printing technology would completely replace traditional building techniques. It is possible that it will play a supporting role to a greater extent, especially when creating elements distinguished by unusual and complex geometry [21,70]. This requires the use of an appropriate building material with appropriate technical properties, thanks to which a sufficiently good print quality is achieved [21,31].

The combination of conventionally separate processes: formwork preparation, reinforcement preparation, and casting of concrete—into one automatized production process, can result in waste-free, material-efficient, and geometrically unlimited methods of producing complex concrete structures [51]. The 3D printing of plastics creates a unique opportunity to efficiently recycle plastic waste available all over the world [54]. Harnessing the 3D printing of plastic is currently underrated in civil engineering. As described in the paper, 3D printed spatial plastic elements have the potential to significantly influence the construction process. Using them for the creation of lost formworks or non-conventional reinforcement is feasible. In the future, steel rebars will be used only for critical and most loaded parts of concrete structures. All other types of reinforcement (secondary structural rebars, stirrups, meshes, etc) will be substituted by 3D printed plastic spatial elements.

## Data Availability

The data presented in this study are available on request from the corresponding author.

## Appendix A

**Table A1:** Summary of the results.

Bibliography Item	Property	Value	Unit
[53]	Compressive strength mortar	22.7	MPa
	Flexural strength mortar	4.3	MPa
	Splitting tensile strength mortar	2.8	MPa
	Apparent density mortar	2040	kg/m^3^
	Dynamic modulus of elasticity mortar	32.0	GPa
	Column strain (depening on the cross-section)	1.7–3.5	%
	Compressive strength columns—full area of a cross-section (depening on the cross-section)	16.5–24.7	MPa
	Compressive strength columns—only area of concrete (depening on the cross-section)	20.9–28.4	MPa
	The energy needed to obtain the deformation at the achievement of the maximum force of columns (depening on the cross-section)	18.3–51.0	J
[54]	Compressive strength cement matrix	45.6	MPa
	Flexural strength matrix	7.0	MPa
	Flexural strength of formwork—matrix element with no ribs	12.8	MPa
	Flexural strength of formwork—matrix element with 3 ribs of 15 mm	30.4	MPa
[55]	Compressive strength mortar	43.3	MPa
	Flexural strength mortar	8.0	MPa
	The amount of energy needed to obtain the maximum bending force	from 244 (for H = 5 mm and d = 1.00 mm) to 7049 (for H = 20 mm and d= 2.00 mm)	Nmm
	Flexural toughness of tested prism specimens dor l/150	700–1300	Nmm
	Flexural toughness of tested prism specimens dor l/75	2500–4500	Nmm
[57]	The flexural strength of plain mortar	5.8	MPa
	The flexural strength for reinforced samples	<5.8	MPa
	Obtained deflection for the plain mortar	<0.1	mm
	Obtained deflection for the reinforced samples	0.6–1.0	mm
	Ductile failure strength	0.0—for the plain mortar; 2.74–3.69 for the reinforced samples	MPa
	Normalized ductile failure strength	0.0—for the plain mortar; 3.31–5.56 for the reinforced samples	MPa
	Total work	0.07—for the plain mortar; 0.59–0.91 for the reinforced samples	J
	Normalized total work	0.07—for the plain mortar; 30.34–45.52 for the reinforced samples	J/cm^3^
[58]	Peak load	0.77 (randomness 0.1); 0.66 (randomness 0.5)	kN
	Slope	2.06 (randomness 0.1); 1.57 (randomness 0.5)	kN/mm
[60,61,62]	Flexural strength	4.58 (reference sample); 4.69–6.30 (reinforced samples)	MPa
	Deflection capacity	0.36 (reference sample); 0.34–5.55 (reinforced samples)	mm
	Tensile strength	3.14 (reference sample); 2.42 (reinforced sample ST)	MPa
	Strain capacity	0.02 (reference sample); 0.58 (reinforced sample ST)	%
[63]	Flexural strength of reinforced samples (depends on the type of reinforcement)	2.30–3.04	MPa
	Deflection capacities (depends on the type of reinforcement)	0.05–0.58	mm
	Peak toughness (depends on the type of reinforcement)	15.92–652.37	Nmm
[64]	Deflection	<0.5 (reference sample); 2.5–4.00 (reinforced sample)	mm
	Maximum load	0.6 (reference samples); 0.45–0.55 (with polymeric fibers); 1.1–1.6 (with metallic fibers)	kN
	Thougness index	2.87–3.12 (with polymeric fibers); 41.63–51.15 (with metallic fibers)	-
[65]	First crack load	2056 for beams with PLA; 1553 for beams with PETG	N
	Max load	3296 for beams with PLA; 6321 for beams with PETG	N
	Max strength	5.9 for beams with PLA; 11.4 for beams with PETG	MPa
	Energy	16.3 for beams with PLA; 91.6 for beams with PETG	J

## References

[1] Bose S., Vahabzadeh S., Bandyopadhyay A. (2013). Bone tissue engineering using 3D printing. Mater. Today.

[2] McCullough E.J., Yadavalli V.K. (2013). Surface modification of fused deposition modeling ABS to enable rapid prototyping of biomedical microdevices. J. Mater. Process. Technol..

[3] Kothman I., Faber N. (2016). How 3D printing technology changes the rules of the game Insights from the construction sector. J. Manuf. Technol. Manag..

[4] Huang S.H., Liu P., Mokasdar A., Hou L. (2013). Additive manufacturing and its societal impact: A literature review. Int. J. Adv. Manuf. Technol..

[5] Tay Y.W.D., Panda B., Paul S.C., Noor Mohamed N.A., Tan M.J., Leong K.F. (2017). 3D printing trends in building and construction industry: A review. Virtual Phys. Prototyp..

[6] Gibson I., Rosen D.W., Stucker B. (2010). Additive Manufacturing Technologies: Rapid Prototyping to Direct Digital Manufacturing.

[7] Ghaffar S.H., Corker J., Fan M. (2018). Additive manufacturing technology and its implementation in construction as an eco-innovative solution. Autom. Constr..

[8] Gebler M., Schoot Uiterkamp AJ M., Visser C. (2014). A global sustainability perspective on 3D printing technologies. Energy Policy.

[9] Gebhard L., Mata-Falcón J., Anton A., Dillenburger B., Kaufmann W. (2021). Structural behaviour of 3D printed concrete beams with various reinforcement strategies. Eng. Struct..

[10] Hager I. (2018). Rewolucja technologiczna w budownictwie—Druk 3D budynków i obiektów inżynieryjnych. Ujęcie Aktualnych Problemów Budownictwa.

[11] Hager I., Golonka A., Putanowicz R. (2016). 3D Printing of Buildings and Building Components as the Future of Sustainable Construction?. Procedia Eng..

[12] Ngo T.D., Kashani A., Imbalzano G., Nguyen K.T., Hui D. (2018). Additive manufacturing (3D printing): A review of materials, methods, applications and challenges. Compos. Part B Eng..

[13] Kreiger M.A., Mulder M.L., Glover A.G., Pearce J.M. (2014). Life cycle analysis of distributed recycling of post-consumer high density polyethylene for 3-D printing filament. J. Clean. Prod..

[14] Ma G., Li Z., Wang L. (2018). Printable properties of cementitious material containing copper tailings for extrusion based 3D printing. Constr. Build. Mater..

[15] Choi J.W., Medina F., Kim C., Espalin D., Rodriguez D., Stucker B., Wicker R. (2011). Development of a mobile fused deposition modeling system with enhanced manufacturing flexibility. J. Mater. Process. Technol..

[16] Berman B. (2012). 3-D printing: The new industrial revolution. Bus. Horizons.

[17] Kruth J.-P., Leu M., Nakagawa T. (1998). Progress in Additive Manufacturing and Rapid Prototyping. CIRP Ann..

[18] Wong K.V., Hernandez A. (2012). A Review of Additive Manufacturing. ISRN Mech. Eng..

[19] Kret W., Michnowicz M. (2013). Drukarka 3D oparta na dokumentacji. Eduk. Tech. Inform..

[20] Mehar P., Khobragade P., Mendhe M., Bhada S., Singh V., Salodkar P. (2020). 3d Printing Trends in Building and Construction Industry. Int. J. Sci. Res. Sci. Technol..

[21] Labonnote N., Rønnquist A., Manum B., Rüther P. (2016). Additive construction: State-of-the-art, challenges and opportunities. Autom. Constr..

[22] Major M., Minda I. (2016). The use of 3D printing technologies in the civil engineering. Zesz. Naukowe Politech. Częst. Ser. Bud..

[23] Javelin—A TriMech Company. https://www.javelin-tech.com/3d/industry/architecture/.

[24] The World’s First Office from a 3D Printer Ringier Axel Springer Polska Sp. z o.o. Członek Izby Wydawców Prasy i Związku Kontroli Dystrybucji Prasy. https://www.forbes.pl/life/wydarzenia/biuro-wydrukowane-drukarka-3d/jk9elx7.

[25] Dodziuk H. (2020). Druk 3D w budownictwie. Napędy Sterow..

[26] 10 Completely 3D Printed Houses Appear in Shanghai, Built under a Day. http://www.3ders.org/articles/20140401-10-completely-3d-printed-houses-appears-in-shanghai-built-in-a-day.html.

[27] Wheeler A. http://3dprintingindustry.com/2015/01/19/winsun-3d-printing-building/.

[28] Wang L. https://inhabitat.com/chinese-company-builds-3d-printed-villa-inless-than-3-hours/.

[29] Mendoza H.R. (2016). https://3dprint.com/138336/syska-hennessy-dubai-office/.

[30] Scott C. (2018). https://3dprint.com/220965/sand-3d-printing-smart-slab/.

[31] Rębosz-Kurdek A., Gierulski W., Szmidt A. (2018). The innovative 3D printer head. Part 1. Concept. Mechanik.

[32] Drury C. (2018). https://www.independent.co.uk/news/world/europe/3d-printed-home-world-first-france-a8298446.html.

[33] Boffey D. (2018). https://www.theguardian.com/artanddesign/2018/jun/06/netherlands-to-build-worlds-first-habitable-3d-printed-houses.

[34] Cheniuntai N. (2019). https://www.apis-cor.com/dubai-project.

[35] Gadomska H. (2020). Największy na Świecie Budynek z Drukarki 3D Stanął Właśnie w Dubaju. https://www.focus.pl/artykul/najwiekszy-na-swiecie-budynek-z-drukarki-3d-stanal-wlasnie-w-dubaju.

[36] Usidus M. Will 3D Printing Revolutionize Construction?. https://mlodytechnik.pl/technika/28916-czy-druk-3d-zrewolucjonizuje-budownictwo.

[37] Howard D. (2013). https://www.dezeen.com/2013/06/03/silkworms-and-robot-work-together-to-weave-silk-pavilion/.

[38] Wu P., Wang J., Wang X. (2016). A critical review of the use of 3-D printing in the construction industry. Autom. Constr..

[39] Zhang J., Wang J., Dong S., Yu X., Han B. (2019). A review of the current progress and application of 3D printed concrete. Compos. Part A Appl. Sci. Manuf..

[40] Malik Chua J. (2017). World’s First 3D-Printed Pedestrian Bridge Pops up in Madrid. Inhabitat.

[41] Apis cor Company Dubai Project. http://apis-cor.com/en/about/blog/features-and-perspectives-of-3d--printing.

[42] IAAC (2017). Large Scale 3D Printing. https://iaac.net/research-projects/large-scale-3d-printing/3d-printed-bridge.

[43] Buchanan C., Gardner L. (2019). Metal 3D printing in construction: A review of methods, research, applications, opportunities and challenges. Eng. Struct..

[44] Niu J., Choo H.L., Sun W., Mok S.H. (2018). Numerical study on load-bearing capabilities of beam-like lattice structures with three different unit cells. Int. J. Mech. Mater. Des..

[45] Strauss H., Emmer Pfenninger Partner A.G., Knaack U. (2015). Additive manufacturing for future facades: The potential of 3D printed parts for the building envelope. J. Facade Des. Eng..

[46] Galjaard S., Hofman S., Ren S. (2015). New Opportunities to Optimize Structural Designs in Metal by Using Additive Manufacturing. Advances in Architectural Geometry 2014.

[47] Wangler T., Lloret E., Reiter L., Hack N., Gramazio F., Kohler M., Bernhard M., Dillenburger B., Buchli J., Roussel N. (2016). Digital Concrete: Opportunities and Challenges. RILEM Tech. Lett..

[48] Wangler T., Roussel N., Bos F.P., Salet T.A.M., Flatt R.J. (2019). Digital Concrete: A Review. Cem. Concr. Res..

[49] Gosselin C., Duballet R., Roux P., Gaudillière N., Dirrenberger J., Morel P. (2016). Large-scale 3D printing of ultra-high performance concrete—A new processing route for architects and builders. Mater. Des..

[50] https://bce.au.dk/en/research/examples-of-research-projects/recycled-plastics-transformed-into-3d-printing-material/.

[51] Hack N., Lauer W.V. (2014). Mesh-mould: Robotically fabricated spatial meshes as reinforced concrete formwork. Archit. Des..

[52] Hack N., Lauer W.V., Gramazio F., Kohler M. Mesh Mould: Differentiation for enhanced performance. Proceedings of the Rethinking Comprehensive Design: Speculative Counterculture—Proceedings of the 19th International Conference on Computer-Aided Architectural Design Research in Asia (CAADRIA).

[53] Katzer J., Skoratko A. (2021). Concept of using 3D printing for production of concrete-plastic columns with unconventional cross-sections. Materials.

[54] Katzer J., Szatkiewicz T. (2019). Properties of concrete elements with 3-D printed formworks which substitute steel reinforcement. Constr. Build. Mater..

[55] Katzer J., Szatkiewicz T. (2020). Effect of 3D Printed Spatial Reinforcement on Flexural Characteristics of Conventional Mortar. Materials.

[56] Song G.H., Jing S.K., Zhao F.L., Wang Y.D., Xing H., Zhou J.T. (2017). Design Optimization of Irregular Cellular Structure for Additive Manufacturing. Chin. J. Mech. Eng. Engl. Ed..

[57] Xu Y., Zhang H., Gan Y., Šavija B. (2021). Cementitious composites reinforced with 3D printed functionally graded polymeric lattice structures: Experiments and modelling. Addit. Manuf..

[58] Xu Y., Zhang H., Šavija B., Chaves Figueiredo S., Schlangen E. (2019). Deformation and fracture of 3D printed disordered lattice materials: Experiments and modeling. Mater. Des..

[59] Yazdani Sarvestani H., Akbarzadeh A.H., Niknam H., Hermenean K. (2018). 3D printed architected polymeric sandwich panels: Energy absorption and structural performance. Compos. Struct..

[60] Xu Y., Šavija B. (2019). Development of strain hardening cementitious composite (SHCC) reinforced with 3D printed polymeric reinforcement: Mechanical properties. Compos. Part B Eng..

[61] Savija B. (2020). Use of 3D printing to create multifunctional cementitious composites: Review, challenges and opportunities. RILEM Tech. Lett..

[62] Xu Y. Creating strain hardening cementitious composites (SHCCs) through use of additively manufactured polymeric meshes as reinforcement. Proceedings of the 10th International Conference on Fracture Mechanics of Concrete and Concrete Structures.

[63] Gödek E., Şevik S., Özdilli Ö. A Study on Flexural Behavior of Cement Paste Reinforced by Using 3D-Printed Polylactic Acid-Based Reinforcement. Proceedings of the 2nd International ICONTECH SYMPOSIUM on Innovative Surveys in Positive Sciences.

[64] Farina I., Fabbrocino F., Carpentieri G., Modano M., Amendola A., Goodall R., Feo L., Fraternali F. (2016). On the reinforcement of cement mortars through 3D printed polymeric and metallic fibers. Compos. Part B Eng..

[65] Shweiki A., Junaid M.T., Barakat S. Flexural characteristics of mortar cement reinforced with 3D-printed polymer. Proceedings of the 4th World Congress on Civil, Structural, and Environmental Engineering (CSEE’19).

[66] Cesaretti G., Dini E., De Kestelier X., Colla V., Pambaguian L. (2014). Building components for an outpost on the Lunar soil by means of a novel 3D printing technology. Acta Astronaut..

[67] Paolini A., Kollmannsberger S., Rank E. (2019). Additive manufacturing in construction: A review on processes, applications, and digital planning methods. Addit. Manuf..

[68] Khoshnevis B. (2004). Automated construction by contour crafting—related robotics and information technologies. Autom. Constr..

[69] Cai S., Ma Z., Skibniewski M.J., Bao S. (2019). Construction automation and robotics for high-rise buildings over the past decades: A comprehensive review. Adv. Eng. Inform..

[70] Delgado Camacho D., Clayton P., O’Brien W.J., Seepersad C., Juenger M., Ferron R., Salamone S. (2018). Applications of additive manufacturing in the construction industry—A forward-looking review. Autom. Constr..

[71] Dickson A.N., Abourayana H.M., Dowling D.P. (2020). 3D printing of fibre-reinforced thermoplastic composites using fused filament fabrication—A review. Polymers.

[72] Ahmed W., Alnajjar F., Zaneldin E., Al-Marzouqi A.H., Gochoo M., Khalid S. (2020). Implementing FDM 3D printing strategies using natural fibers to produce biomass composite. Materials.

